# Relating student perceptions of readiness to student success: A case study of a mathematics module

**DOI:** 10.1016/j.heliyon.2020.e05204

**Published:** 2020-11-16

**Authors:** R.L. van der Merwe, M.E. Groenewald, C. Venter, C. Scrimnger-Christian, M. Bolofo

**Affiliations:** a School of Mathematical and Statistical Sciences, North West University, Vanderbijlpark, South Africa; b School of Computer Science and Information Systems, North West University, Vanderbijlpark, South Africa

**Keywords:** Education, Student readiness, Throughput rates, Student preparedness, Higher education, STEM, Mathematics

## Abstract

This study correlates the readiness survey scores of bona fide first year university students with their success in a mathematically based first year module. It follows on the need for skilled individuals in the fields of Science and Technology that exists across the globe and is continually becoming the focus of educational institutions world-wide. Similarly, in South Africa, universities were instructed to increase their intake of students in the fields of Science and Technology so as to provide for the technology orientated needs of the country, as well as to increase participation of previously disadvantaged race groups in these fields. In response to this instruction, universities increased the number of students enrolled in Science and Technology fields by 23% in recent years. The challenge is now to ensure that these students exit the university with a suitable degree in the shortest possible time. Statistics published by the South African Council of Higher Education affirms the extent of this challenge faced by universities—only 51% of students enrolled in the fields of Science and Technology complete their 3-year undergraduate degrees, and some of them took as long as 6 years to complete the 3-year degrees. This still leaves 49% of students that either took more than 6 years to complete their degrees, or did not complete their degrees at all. The underlying cause(s) for these failures must be identified and addressed. As a starting point in this discussion, the questions that this study aim to answer are whether first year students are in fact as prepared for the challenges at university as they perceive themselves to be; and whether student readiness (or lack thereof) can be a root cause for the low throughput rates. This study determines how prepared first year students, at a leading South African university, perceive themselves to be for the demands of university and, specifically, how their perceptions of their readiness in different areas correlate with their academic success in a mathematically based module. The correlation is determined by analysing data gathered through a readiness survey that is completed by first year students at the beginning of the academic year, and their final mark in the mathematics based first year module. The survey is a standardised, self-evaluation tool originally developed by the University of Pretoria. It is also applied at the university where this study is conducted. The survey measures the preparedness of students in different areas and the empirical study shows that there is a statistically significant correlation between the perception of the students regarding their planning ability and the final mark obtained in the mathematics based module.

## 1 Introduction

The Science, Technology, Engineering and Mathematics (STEM) fields play a crucial role in the global economy; STEM entails, for example, critical technological origination, design and problem solving (Wang et al., 2017). These dimensions are indispensable in a modern society; accordingly, global economies progressively depend on the ground-breaking new advances brought along by continued scientific discoveries and technological changes that are effectuated by STEM initiatives (Mandell et al., 2019). It is thus important to educate and up skill people in these fields to address the demands of the modern world. STEM has, therefore, been the focus of educators worldwide; and educational institutions continuously aim to realise the necessity to address the need for skilled graduates in STEM fields (Wang et al., 2017).

Similarly, the South African government identified the growing need for STEM graduates; they also identified a dire need for equality (to overcome the always present socio-economic and digital divide causing social, economic and technological divisions in South Africa) and accordingly instructed stakeholders in the South African Higher Education landscape to transform urgently (DoE, 1997). The main aim of this instruction was to address and overcome inequalities of the past, which were in part created by the apartheid policy of the previous government. However, after assessing the state of the South African Higher Education system in 1997, a document was drafted and distributed to all relevant stakeholders—White Paper 3 indicated that it was crucial to increase and broaden involvement at Higher Education Institutions, as well as to meet the STEM related needs of the economy, by producing more graduates skilled in the fields of STEM. However, involved stakeholders realised that to simply increase the number of students at tertiary institutions to afford equal opportunity to all gender and race groups will not reduce the effects of the divide sufficiently; in addition, to advance the economy the needs of industry must also be sufficiently addressed in the process. So, in response to the white paper instruction, the Government then drafted the National Plan for Higher Education (2001).

The National Plan for Higher Education's aim was mainly to provide and advance equal, all-inclusive educational opportunities to the entire South African population, without any discrimination. However, to realise this, a secondary aim had to be set—its goal was therefore supplemented by a strategic objective to produce more graduates with (STEM) skills and competencies, as are currently needed by the country. Accordingly, priorities were set to increase the number of participants in STEM programmes, as well as to produce graduates with these skills and competencies, as required. The total intake in STEM programmes, at public universities in South Africa, increased over the past years (DHET, 2013a; 2013b; 2014; 2015; 2016; 2017; 2018; 2019). Unfortunately, very few of the students that enrolled for studies in STEM fields completed their degrees, and throughput rates remain low (Council of Higher Education, 2018; 2019).

This study aims to investigate a potential cause of the problematic low throughput rates at tertiary institutions. The researchers focused on a single STEM related module for this initial investigation. They agree with Alvesson and Willmott (1992) that radical and grandiose understanding and subsequent change is not practically achievable in a short space of time; still, consecutive small projects, applied as exploratory case studies such as the one discussed in this paper, is valuable and helps to answer, in time, integrated and more complex questions in a larger context. Mathematics is one of the core dimensions of STEM. The researchers therefore chose a single mathematics based module to start exploring a potential cause for low throughput rates.

This paper is structured as follows: Section 2 gives a brief overview of the Higher Education landscape in terms of (problematic) enrolment versus throughput rates in all fields of study, including STEM fields, in South Africa. It also discusses possible causes of low throughput rates and interventions, as per the literature. Section 3 discusses the research problem addressed by this study. The research approach followed is discussed in Section 4. Section 5 discusses the results of the empirical study. A discussion of the results follows in Section 6 with the limitations of the study given in Section 7. The paper ends off with a conclusion in Section 8.

## 2 Literature review

This section discusses literature that relates to enrolment versus throughput rates at South African Higher Education institutions in all programmes, including STEM programmes. It illustrates that, even though enrolment increases, throughput rates continue to decrease. This is problematic, especially in light of the fact that the country, as the rest of the world, is in dire need of suitably qualified STEM graduates (Wang et al., 2017). This section thus emphasises the importance of this study—it demonstrates that something must be done urgently to timeously deliver more graduates, as is requested by the government, and also needed by industry and for positive economic growth. Therefore, available enrolment versus graduation statistics, as well as possible causes of low throughput rates, and avenues explored to improve it, are discussed next.

### 2.1 Higher education in South Africa

As indicated earlier, a key aim of the government's National Plan was to make higher education more accessible to all South Africans, and increase inclusive enrolments at public universities across the board. This aim was achieved. Statistics published annually by the Department of Higher Education and Training (DHET) (DHET, 2013a; 2013b; 2014; 2015; 2016; 2017; 2018; 2019), shown in Table 1, indicates that South African universities stepped up, and the total intake of students in all programmes increased by 16% from 2010 to 2017; the enrolments in STEM programmes increased by 23% over the same period of time.

**Table 1:** Number of enrolments at Public Higher Education Institutions.

Year	Total enrolments	STEM enrolments	% growth from 2010 (Total)	% growth from 2010 (STEM)
2010	892 925	251 334		
2011	938 200	246 447	5,07%	-1,94%
2012	953 373	273 282	6,77%	8,73%
2013	983 698	283 622	10,17%	12,85%
2014	969 165	287 221	8,54%	14,28%
2015	985 212	294 935	10,34%	17,35%
2016	975 837	295 383	9,29%	17,53%
2017	1 036 984	310 115	16,13%	23,39%

Unfortunately, increased enrolments did not increase the number of graduates significantly, as per the ultimate aim to produce more qualified individuals for economic growth, etc. This is evident from the statistics published by the DHET—shown in Table 2, which indicates the percentage of the students enrolled in a year, versus those that graduate in that same year. The graduation rate between 2010 and 2017 of undergraduate students in all fields at public universities in South Africa has never risen above 21% (DHET, 2013a; 2013b; 2014; 2015; 2016; 2017; 2018; 2019). From this, it is thus clear that graduation rates in all programmes, including STEM, remain problematically low (DHET, 2013a; 2013b; 2014; 2015; 2016; 2017; 2018; 2019).

**Table 2:** Number of graduates Public Higher Education Institutions.

Year	Total graduates	STEM graduates	Graduation rate (Total)	Graduation rate (STEM)
2010	153 325	42 760	17,2%	17,0%
2011	160 617	46 100	17,1%	18,7%
2012	165 986	48 848	17,4%	17,9%
2013	180 823	53 176	18,4%	18,7%
2014	185 385	55 577	19,1%	19,3%
2015	191 524	58 090	19,4%	19,7%
2016	203 076	59 125	20,8%	20,0%
2017	210 931	61 581	20,3%	19,9%

In addition, statistics published by the Council of Higher Education (CHE) also shows that the success rate (as indicated by the percentage of modules passed in a year, taking into account the number of credits of said modules) for undergraduate courses (including STEM courses) never exceeded 79% between the years 2011 and 2017—see Table 3 (Council of Higher Education, 2019).

**Table 3:** Course success rate at Public Higher Education Institutions.

Year	2011	2012	2013	2014	2015	2016	2017
Course success rate	76%	77%	74%	78%	79%	79%	78%
Course success rate in STEM	74%	75%	77%	76%	78%	79%	77%

According to the CHE only 29% of the students enrolled for a 3-year degree in 2011, as well as in 2012, completed the degree in the minimum 3-year time frame (Council of Higher Education, 2018; 2019). In the fields of Science and Technology the throughput rate is even lower, since only 21% of the students enrolled in 2011, and 22% of the students enrolled in 2012, completed the 3-year degree in the minimum time. For enrolments in the fields of STEM, in both 2011 and 2012, a mere 51% of the students completed the 3-year degree after a total of 6 years (see Tables 4 and 5).

**Table 4:** Throughput rates at Public Higher Education Institutions for students enrolled for the first time in 2011 for a 3-year degree (excluding UNISA).

Year	% graduated (Total)	% graduated (STEM)
2013	29%	21%
2014	18%	18%
2015	8%	9%
2016	3%	3%
Total	58%	51%

**Table 5:** Throughput rates at Public Higher Education Institutions for students enrolled for the first time in 2012 for a 3-year degree (excluding UNISA).

Year	% graduated (Total)	% graduated (STEM)
2014	29%	22%
2015	18%	18%
2016	8%	8%
2017	3%	3%
Total	58%	51%

The statistics discussed here emphasises the extent of the challenge faced by Higher Education Institutions to deliver the much needed graduates. It is evident that avenues must be explored to improve the throughput and graduation rates at Higher Education Institutions. So, avenues previously explored, as per the existing literature, are discussed next.

### 2.2 Avenues investigated to improve graduation rates

Low graduation rates are not a problem that is unique to South Africa. The question pertaining to why some students succeed and others fail, led to a plethora of research that was done in an attempt to determine the factors affecting the success of students entering the Higher Education environment. Researchers across the globe have been attempting to find a solution to this universal problem.

Some of the investigations cited in the literature focussed on the wellbeing of students; refer for example to the study by Phan et al. (2019), who attempted to predict and enhance positive emotions among students in Taiwan. Ngalo-Morrison (2017) also suggests that more focus should be placed on positive feedback and success stories, rather than on failure and negative feedback. In a study by Anderson (2017) on the loneliness of female students in the field of STEM it was found that social support is very important. However, this would not necessarily resolve the issue at hand, i.e. improve graduation rates of current and future students, in a third world country, with a large digital divide and huge socio economic gaps in the diverse population.

Fakude (2012) found that external factors such as financial difficulties, enrolment issues, political affiliations and availability of lecturers were some of the factors affecting performance of students at a South African university. Petersen et al. (2009), on the other hand, indicate that psychological factors play a pivotal role in the student's ability to adjust to the tertiary environment. Fraser and Killen (2005) also investigated the factors influencing performance at two South African universities and found that successful students tend to be self-motivated and hardworking, and has the ability to work independently, while unsuccessful students generally lack self-discipline, time management skills and do not have good study techniques. Malefo (2000) investigated psycho-social factors and found that female African students at a university in South Africa with a family environment in which the parents were the figures of authority tend to be more successful, and students with less stress coped better since they exercised problem focussed efforts to seek solutions to problems. Chen and Kelly (2013) identified sufficient financial support, personal academic assistance, social support, and a positive campus environment as important factors for the retention of STEM students. According to Schulz (2008) there are also other important areas and soft skills that contribute to the success of students, such as communication skills, critical thinking and creativity. Cromley et al. (2016) identified various factors of motivation, such as self-confidence and remaining focussed on classes and studying to play a large role in the successful completion of studies in the fields of STEM.

Many of the investigations cited in the literature were focussed on finding early warning signs that could predict potential failure. As an example, a study done by Murray (2014) found that students receiving sufficient financial aid and residence-based accommodation graduated in a shorter period of time. The study of Adekitan and Salau (2019) explored the performance of Nigerian engineering students in the first three years of study in an attempt to predict their grades in the 5th year of study. Adekitan and Salau (2019) found that the graduating results of engineering students in Nigeria can be reasonably predicted by their performance in the first three years of their study. Sithole et al. (2017) reviewed some of the factors playing a role in the retention of STEM students, such as mathematical proficiency, study habits and academic engagement, time management and motivation and self-efficacy. He found that the presentation of orientation programmes, mathematics review sessions and creating learning communities among students are some of the strategies to improve retention. He also emphasized the importance of having systems in place to detect at risk students early.

In many of the studies the effect of interventions were investigated as well. Du Preez et al. (2008) and Agherdien (2014) both found that interventions, after identifying relevant gaps, had a positive effect on performance. Du Preez et al. (2008) suggest the rethinking of learning material in mathematics to meet students where they are, since lecturers cannot commence a module at a knowledge level where they assume students should be. Du Preez et al. (2008) further suggest that students should be actively involved in learning content and that learning facilitation should also be reconsidered. According to Agherdien (2014), some American schools use a scaffolding approach which requires students to gradually work independently; and group work is used to maximise student engagement. Agherdien (2014) further indicates that certain South African universities use bridging programmes, (which includes a soft skills component, e.g. time-management, and a technical component, e.g. additional mathematical modules), orientation programmes, student counselling, tutoring and mentorship programmes to address student readiness. Despite all these attempts the systematic review of van den Hurk et al. (2019), on the effectiveness of STEM related interventions, shows that there are still a great need for more research on these interventions.

The background against which this study was done is informed by the investigations on the readiness or preparedness of students entering the university. Both Hourigan and O'Donoghue (2007) and Agherdien (2014) claim that the preparedness of the students is largely influenced by the type of schooling that they received. Hourigan and O'Donoghue (2007) further suggest that the exam focussed teaching in schools in Ireland leads to many gaps in the prior knowledge of the university students. Agherdien (2014) found that variables such as course and student fit, study methods, self-management, reading, writing, subject proficiency, psychological and social adjustment, financial support, personal circumstances and student engagement affect student readiness. In their study Du Preez et al. (2008) gave possible reasons for the students' low level of preparedness as being: inadequate schooling, poorly qualified school teachers, coaching for the final school examination and inadequate content of the school syllabus. These issues must be resolved early on so as to ensure success later on in students' academic endeavours.

Some of the investigations into the performance of students were based on the students’ own perceptions. One such a study was done by Tuckman (1992), who investigated the importance of planning on motivation. Tuckman grouped students according to their own perceptions of their competence and found that if he provided the students with a planning strategy to complete the task their performance improved, especially for those who rated themselves low on competence. In a study done by Ochse (2003), he compared the perceptions students had regarding their ability with their actual performance. He found that the better they perceived themselves to be the more unrealistic they were. Students that perceived themselves to be weak were more realistic. Students scoring themselves low are most likely the ones that will struggle. On the other hand, students scoring themselves high could also be struggling.

So, overall, research indicates that students are not always ready for the challenges posed to them at university. It also indicates that timely intervention can be a solution to the pass rate problem. It is thus crucial to identify at risk students, in terms of their readiness to positively adapt to a university environment, and the interventions required to assist them. This must be done as early as possible in order to start with the intervention timeously. The problem statement is posed next.

## 3 The problem statement

Given the low graduation rates, universities would like to ensure that all the students that enrol in the first year, complete their degree in the shortest possible time. It is thus important to deliver quality education and have adequate support available during the student's studies. However, even with quality education and effective support, students will still struggle if they were not prepared for the challenges the tertiary environment brings. Hence, the research question posed in this study: *“Are first year STEM students really as prepared for the challenges at university as they perceive themselves to be?”*

This study builds on the premise that at risk students must be identified as early as possible, since this may increase throughput rates of programmes. The researchers build on the premise that a student's perception of his/her readiness may be an indication of the students' ability to succeed later on. As a further investigation on how to identify at risk students, already at the beginning of their first year, and offer the needed support as soon as possible to be able to deliver graduates in the shortest possible time, this study correlated the perceived preparedness of students for university, with the final mark of these students in a specific mathematics based first year module offered at a leading university in South Africa (hereafter to referred to in this paper as “the university”). The researchers focused on a mathematics based module, as a subset of STEM, for the initial investigation discussed in this paper. The research approach followed is discussed next.

## 4 The research approach

This study is conducted from a positivistic point of view, where the assumption is made that reality can be observed objectively and can be described by measurable properties. The drawing of inference about a certain phenomenon in a sample to a population is evidence of a positivistic view (Myers, 1997). It is suitable for this study because the researchers wanted to observe physical phenomena that occurred, and draw conclusions from the survey data gathered and analysed. The instrument to gather data, the data gathered and the method of analyses as well as the results obtained are discussed next.

### 4.1 The instrument: the STARS survey

In 2015 the University of Pretoria (UP), based in South Africa, developed a tool called the Student Academic Readiness Survey (STARS) to measure how prepared first year students perceive themselves to be for the academic programme ahead of them. The aim of this survey was to identify at risk students at the beginning of the first academic year and offer the needed support as soon as possible. UP requests all their first year students to complete the survey at the beginning of the academic year before any classes start. The university where this study was conducted, followed suit and also requested all first year students to complete the STARS survey to assess their perceived readiness.

The handbook to the STARS results defines the seven areas evaluated with the STARS survey and these areas, with the factors evaluated within each area, are shown in Table 6 (Lemmens, 2015). The development and validation of the questionnaire used in this survey is discussed in the thesis of Lemmens (2011).

**Table 6:** Areas and factors measured with the STARS survey.

Area evaluated	Factor evaluated	Description of factor
Motivational factors	Planning	The students' ability to plan their studies by setting target goals for learning.
	Locus of control	The level of personal responsibility that students take for their actions.
	Self-efficacy	The students' perception of their ability to achieve academic goals.
	Leadership	The students' perception of their leadership ability.
Academic involvement	Test taking skills	The perception of the students' ability to be free of anxiety during tests and their levels of concentration during tests.
	Engagement	The level to which students anticipate to be involved with their studies and lectures.
	Pleasure reading	The level to which students find pleasure in reading to determine their attitude towards reading and reading behaviour.
Well-being	General well-being	The perception of students' emotional and physical health.
Integration and support	Institutional support	The sufficiency of information from the university before enrolling to help students make decisions regarding their choice of institution and programme.
	Financial support	The level of financial support that students have for their studies.
	Family support	The level of family support that students have during their studies.
	Sociability	The extent to which students can relate easily to others.
Vocational identity	Career Exploration	The extent to which students believe that their programme is related to their interests and career choice.
	Career guidance	The extent to which career guidance was sought after.
Academic skills needed	Study skills	The level to which students are able to study independently.
	Reading skills	The level to which students are able to read.
	Writing skills	The students' ability to write.
	Time management skills	The ability of students to manage their time.
	Mathematics skills	The level at which students' can work with numbers.
	Computer skills	The level of students' computer knowledge.
	Presentation skills	The ability of students to talk in front of people and present.
Goal orientation	Goal achievement	The belief in your ability to find ways to solve problems and achieve your goals.
	Future vision	The level to which you are optimistic based on the belief that the future holds promise and your goals will be reached.
	Hope agency	The ability to imagine actions and behaviour to reach your goals.
	Hope pathway	The ability to find ways or routes to your goals.
	Optimism	The level to which you expect good things to happen to you.
	Self-motivation	The level to which you take responsibility and action.
	Hopefulness	The level to which you are positive about the future.
	Agency	The ability to formulate goals and work toward them.

So, the STARS instrument is also applied to all first year students at the university where this study was conducted. The researchers decided to use data gathered using this tool for this study, as the tool was already validated and in use at this university. They expected to (and did) learn about the students’ perceived readiness for university from this.

The students’ responses in the survey are scored using different scales and accompanied by an interpretation. The scales used for scoring the different factors measured, with the indication for each possible outcome, is given in Table 7.

**Table 7:** Scales used for scoring the factors measured and their indication.

Factors measured	Scale	Score	Indication
Career guidance Academic skills needed	1–3	1	Student needs no assistance
		2	Student needs some assistance
		3	Student needs a great amount of assistance
Future vision	1–7	1–3	Low score
		4–5	Average score
		6–7	High score
Leadership Hope agency Hope pathway Optimism	1–9	1–4	Low score
		5–6	Average score
		7–9	High score
Goal achievement Self-motivation Planning Locus of control Hopefulness Agency Self-efficacy Test taking skills Sociability Pleasure reading Engagement General well-being Institutional support Financial support Family support Career explorations	1–10	1–4	Low score
		5–7	Average score
		8–10	High score

### 4.2 Data collection and analysis approach

As indicated earlier, the university in this study soon followed UP and started using the STARS survey to evaluate the perceived readiness of the students entering their university. During orientation, at the beginning of the academic year, bona fide first year students are requested to complete the readiness survey. Completion of it is voluntarily. Indication by the students whether the data gathered using this survey may be used for research purposes, is also voluntary. The survey is completed at the beginning of the academic year before any tests are written and before students attended any classes.

In this study the researchers used data of completed surveys and compared the results in each of the areas measured in the STARS survey with the final marks obtained by the students in a particular mathematics based first year module, in order to determine whether there is any correlation between the factors measured by the STARS survey, and the marks obtained in this module. This particular module consists of a theoretical component with applications and a practical component that consists of various small assignments that eventually contribute to a large project. This indicated to the researchers whether the student's perception of his/her own readiness, in each of the measured areas, was proven to be accurate, when compared to his/her success in the module.

#### 4.2.1 Sample and data collection

To ensure that the study complies with all the regulations of the University an application for approval was submitted to the Research Gatekeeper. After receiving approval for the study from the Research Gatekeeper of the university (North-West University research gatekeeper committee reference number: NWU-GK-2018-20) the quantitative data, of the students that gave written consent, for this study were obtained from the central intelligence unit of the university. It comprised of the STARS results as well as participation marks and exam results in the particular module. The survey is voluntary and aims to identify possible areas where the university can support students. The total number of respondents in the study was 66 (Males = 54.55%, Females = 45.45%) from a group of 360 students. The group were all students from the same programme, with Mathematics and Statistics as majors.

The dependent variable in this study is the final mark (FM) in a particular mathematics based module which is also one of the majors for this degree. The final mark is calculated using the participation mark and the exam mark. The independent variables in this study, given in Table 8, are the factors measured with the STARS survey. After completing the survey, the student received a score for each of these factors.

**Table 8:** Independent variables and abbreviations.

Independent variables	Abbreviation	Independent variables	Abbreviation
Planning	PL	Locus of control	LOC
Self-efficacy	SE	Leadership	LS
Test taking skills 1	TTS1	Engagement	EN
Reading behaviour	RB	General well-being	GWB
Institutional support	IS	Financial support	FIS
Sociability	SOC	Career exploration	CE
Study skills	SS	Reading skills	RS
Writing skills	WS	Time management skills	TMS
Math skills	MS	Computer skills	CS
Presentation skills	PS	Test taking skills 2	TTS2
Family support	FAS		

#### 4.2.2 Data analysis

The data were analysed using stepwise multiple regression to determine which independent variables are linearly related to the dependent variable, and have a statistical significant correlation with the dependent variable. SPSS was used to analyse the data and test the model assumptions. The outcome of the analysis is discussed next.

## 5 Results

Table 9 indicates that the stepwise multiple regression only included one of the original 20 independent variables in the final model. “Planning” was the only independent variable that has a beta value that is significant at a 0.05 significance level (as seen in Table 10) with a p-value of 0.022. The standardised beta coefficient is used to compare the strength of the effect of each individual independent variable to the dependent variable. The higher the absolute value of the standardised beta coefficient, the stronger the effect. The standardised beta coefficient value is 0.282.

**Table 9:** Variables included in regression model.

Model	Variables Entered	Variables Removed	Variables Entered/Removed^a^
			Method
1	Planning		Stepwise (Criteria: Probability of F to enter ≤.050, Probability of F to remove ≥.100).

a Dependent Variable: FinalMark.

**Table 10:** Coefficients.

Model		Coefficients^a^				
		Unstandardized Coefficients		Standardized Coefficients	t	Sig.
		B	Std. Error	Beta		
1	(Constant)	53.080	5.957		8.910	0.000
	Planning	2.074	0.883	0.282	2.348	0.022

a Dependent Variable: FinalMark.

Table 11 shows that the overall fit of the model is poor, with an R^2^-value of 0.079. This means that this multiple regression model only explains 7.9% of the variation in the final module mark. It is therefore clear that there might be other factors outside of this study that play a role in the success of the student.

**Table 11:** Model summary.

Model	R	Model Summary		
		R Square	Adjusted R Square	Std. Error of the Estimate
1	.282^a^	0.079	0.065	12.566

a Predictors: (Constant), Planning.

Table 12 indicates that there is a statistically significant correlation between the independent variable, planning, and the final mark obtained in the module (r = 0.282, p < 0.05). As a student's score for planning increases, the student's final module mark also increases.

**Table 12:** Correlations.

		Correlations																					
		FM	PL	LOC	SE	LS	TTS1	EN	RB	GWB	IS	FIS	FAS	SOC	CE	SS	RS	WS	TMS	TTS2	MS	CS	PS
FM	Pearson Correlation	1	282∗	-0.021	0.052	-0.029	0.055	0.098	-0.133	-0.055	0.112	0.119	0.015	-0.218	0.121	-0.020	0.008	0.063	-0.189	-0.057	-0.116	0.068	0.040
	Sig. (2-tailed)		0.022	0.867	0.678	0.820	0.658	0.433	0.288	0.661	0.371	0.339	0.904	0.079	0.333	0.872	0.947	0.617	0.128	0.650	0.355	0.590	0.752

The variable “planning” measures the students’ perception about their own ability to plan their studies by setting target goals for learning, and also indicates their belief about competence, success and effort (Lemmens, 2015). None of the other independent variables measured by the STARS survey has any statistically significant correlation with the final mark.

## 6 Discussion

From the results it is clear that the variable with the strongest effect on successful completion of the mathematics module is “planning”. Planning forms part of the motivational factors that are evaluated by the STARS survey. Lemmens (2015) defines planning, in the handbook to the STARS results, as the ability to plan your studies by setting goals. The goals that are measured here are referred to as target goals for learning. The student can evaluate his/her performance with the target goals. More general goals are also measured and indicate the reason a student is pursuing a task. A combination of target goals indicates the specific learning goals that students set, as well as beliefs about ability, competence, success and effort. The scores on the report range from one to ten. Low scores (1–4) thus indicate students’ perceived inability to set target goals. Such a student will also have low beliefs about his/her ability to achieve academically and will invest less effort in their work. Students with average scores (5–7) set intermediate goals and have realistic efficacy beliefs. High scores (8–10) indicate students that set challenging target goals for their studies and would have high efficacy beliefs about their ability to be successful and will invest the necessary effort.

With reference to the sample of students that participated in this study, the implication is that the final mark obtained by them in this mathematics module correlates with their own perceived abilities to plan appropriately, as is indicated by the score that they gave themselves in “planning” prior to commencement of their first academic year. Accordingly, when students scored themselves high in terms of their own perceived ability to plan efficaciously, they were more successful. Consequently, the researchers argue that students that score themselves very low in terms of their own planning skills can (should) be identified and supported to improve their planning skills prior to commencement of formal studies, so as to increase their chances of academic success. The results are consistent with the findings of Tuckman (1992) that planning are important for success. Planning skills are important to determine and manage demanding workloads, as is the case in academic institutions where students participate in challenging programmes, such as STEM programmes, and, in the case of this study, the mathematics based module. Proactively helping them to increase their planning skills can potentially aid to prepare them for the demands of university. It thus makes sense to assist students to increase their planning skills proactively, especially when they indicate that they lack these at the very beginning of their academic years.

This study revealed one of the factors contributing to the low pass rates in this particular module; it enables the university to identify at least a portion of at risk students. The institution can thus intervene prior to commencement of the first academic year. The researchers suggest the following preventative measures: Offering a short course in planning at the beginning of the academic year could eradicate this particular problem and ensure that these students graduate faster, enabling them to enter the workplace soonest. Most universities have student support departments aimed at assisting struggling students—they could conduct the readiness surveys and offer applicable short courses, since they should have (or obtain) the expertise to do so. The Department of Education could also play a role and enhance school learners’ planning skills by introducing planning activities in secondary school and during the teaching of, for example, the Life Orientation subject.

Since the survey is currently voluntary at this university the sample size in this study was very small. If the survey could be made compulsory, and the results from a larger group of students could be analysed, more factors could be identified. Although the overall fit of the model is poor, it is a good starting point for universities to address the perceived and prevalent lack of planning (abilities) in first year students. Once the lack of planning has been eliminated other factors can be investigated further. The researchers also suggest that the study be repeated for other STEM related modules.

## 7 Limitations of the study

This study was done at a small university with relatively small class groups, especially in mathematics related programmes. The incorporation of the STARS survey to the orientation of the first year students have been done for only a few years now, and the participation of the students are still very low, therefore the number of participants in this study is very small. The researchers regard this therefore as a pilot study that leads the way to more research in this field. As per the work of Alvesson and Willmott (1992) that agree that all great ventures start with the first, small step.

Further, this instrument do not address the other important areas and soft skills that contribute to the success of the students (Schulz, 2008). These important areas and soft skills should also be addressed in future. These include, for example, teamwork (i.e. the student's ability to effectively collaborate with other people on a task or project to achieve a common goal); critical thinking (i.e. the student's ability to analyse and understand a problem to reach a solution); organisational skills (i.e. the student's ability to create order and structure to be more productive); and adaptability (i.e. the student's ability to adapt to change in a fast-paced and changing environment).

## 8 Conclusion

This study examined the correlation between students’ perceptions of their readiness for university, and their academic success. In light of the urgent need for more STEM graduates, as identified by the South African government and Higher Education Institutions, it focused on a specific core mathematics (STEM related) module in a Mathematics and Statistics based degree offered at a leading university in South Africa. Literature indicates that enrolment for these have increased, but throughput rates remain low. This is a global issue, but of pertinent importance in the South African context. South Africa is a developing third world country with immense socio-economic and technological digital divides; they have therefore identified a need for equality in education and thus continue to seek to increase the number of STEM graduates to do so.

The study revealed that a low score in students’ perception of their own planning ability correlates with the failure of students in this mathematics based module. So, it may very well be a root cause of low throughput rates and, as such, a good starting point for universities that would like to ensure that all the students that enrol in the first year have a fair chance of completing their degree in the shortest possible time (or at all), would be to address the perceived and prevalent lack of planning (abilities) in first year students. If the survey could be made compulsory and the students that score low in “planning” identified and entered into a workshop on planning, the particular students could be better prepared for the workload and being able to plan their own studies better would ensure that they have a better chance of success. Other areas and soft skills that this instrument did not address, should be addressed in the future. The study should also be extended to include more modules, and more participants.

## Declarations

### Author contribution statement

R. L. Van der Merwe: Conceived and designed the experiments; Performed the experiments; Contributed reagents, materials, analysis tools or data; Wrote the paper.

M. E. Groenewald: Conceived and designed the experiments; Analyzed and interpreted the data; Contributed reagents, materials, analysis tools or data; Wrote the paper.

M. Bolofo: Performed the experiments.

C. Scrimnger-Christian: Analyzed and interpreted the data.

C. Venter: Contributed reagents, materials, analysis tools or data.

### Funding statement

This research did not receive any specific grant from funding agencies in the public, commercial, or not-for-profit sectors.

### Competing interest statement

The authors declare no conflict of interest.

### Additional information

Supplementary content related to this article has been published online at Data In Brief.
